# All-*trans* retinoic acid enhances the effect of 5-aza-2′-deoxycytidine on p16INK4a demethylation, and the two drugs synergistically activate retinoic acid receptor β gene expression in the human erythroleukemia K562 cell line

**DOI:** 10.3892/ol.2014.2133

**Published:** 2014-05-12

**Authors:** LILI XIANG, WEIMIN DONG, RONG WANG, JIANG WEI, GUOQIANG QIU, JIANNONG CEN, ZIXING CHEN, XIAO ZHENG, SHAOYAN HU, XIAOBAO XIE, XIANGSHAN CAO, WEIYING GU

**Affiliations:** 1 Department of Hematology, The First People’s Hospital of Changzhou, Third Affiliated Hospital of Suzhou University, Changzhou, P.R. China; 2 Laboratory of China and United States Cooperation, The First People’s Hospital of Changzhou, Third Affiliated Hospital of Suzhou University, Changzhou, P.R. China; 3 Comprehensive Laboratory, The First People’s Hospital of Changzhou, Third Affiliated Hospital of Suzhou University, Changzhou, P.R. China; 4 Hematology Laboratory, The First People’s Hospital of Changzhou, Third Affiliated Hospital of Suzhou University, Changzhou, P.R. China; 5 Jiangsu Institute of Hematology, The First Affiliated Hospital of Suzhou University, Suzhou, P.R. China; 6 Laboratory of Tumor, The First People’s Hospital of Changzhou, Third Affiliated Hospital of Suzhou University, Changzhou, P.R. China; 7 Department of Hematology and Oncology, Children’s Hospital of Suzhou University, Suzhou, Jiangsu, P.R. China

**Keywords:** K562 cells, 5-aza-2′-deoxycytidine, DNA methylation, all-*trans* retinoic acid, p16INK4a, retinoic acid receptor β

## Abstract

The aim of the current study was to investigate the antineoplastic activities of 5-aza-2′-deoxycytidine (also known as decitabine; DAC) and all-*trans* retinoic acid (ATRA), administered alone or in combination, in K562 cells *in vitro*, as well as the effects on the expression of the tumor suppressor genes, p16INK4a (p16) and retinoic acid receptor β (RAR-β). Cell growth inhibition, differentiation and apoptosis in K562 cells treated with DAC and/or ATRA were detected. The methylation of the p16 and RAR-β genes in the K562 cells was detected using the methylation-specific polymerase chain reaction (PCR) method. Quantitative PCR was used for the detection of the mRNA expression of the p16 and RAR-β genes, and western blot analysis was used to detect protein expression. DAC and ATRA, alone or in combination, had no effect on the growth inhibition, differentiation and apoptosis of the K562 cells. DAC alone induced the demethylation of the p16 gene, and combination of DAC and ATRA demonstrated more evident demethylation of the p16 gene, however, ATRA alone had no effect on methylation. The RAR-β promoter region was not methylated in the K562 cells. DAC in combination with ATRA appeared to produce a greater activation of the RAR-β gene, which led to the upregulation of the RAR-β expression level. ATRA enhanced the effect of DAC on p16 demethylation, and the combination of the two drugs was found to activate RAR-β expression, which indicated that DAC used in combination with ATRA has clinical potential in the treatment of human erythroleukemia.

## Introduction

The regulation of gene expression is contributed to by epigenetic mechanisms that affect the chromatin structure. Aberrant DNA methylation has been found in almost all tumor types, including leukemia (1–3), and is associated with the aberrant silencing of tumor suppressor genes in cancer. However, the demethylation of the promoter-associated CpG islands leads to gene re-expression. 5-Aza-2′-deoxycytidine (also known as decitabine; DAC) is a cytosine nucleoside analogue that shows noteworthy antineoplastic activity in patients with myelodysplastic syndromes (4–6). The antitumor activity of DAC is mediated by the inhibition of DNA methylation, which results in the activation of specific genes. DAC has also been demonstrated to activate the expression of tumor suppressor genes, including p16INK4a (p16) and retinoic acid receptor β (RAR-β), which are silenced by DNA methylation (7–9). All-*trans* retinoic acid (ATRA) is a potent inducer of the granulocytic differentiation of myeloid leukemia cells (10,11) and has been used successfully to treat acute promyelocytic leukemia. However, the antineoplastic effect of the two drugs in combination on acute erythroleukemia has not previously been reported. The current study investigated the antineoplastic activity of DAC and ATRA in the human erythroleukemia K562 cell line *in vitro*, as well as the effects of these agents on the tumor suppressor genes, p16 and RAR-β.

## Materials and methods

### Materials

DAC and ATRA were purchased from Sigma-Aldrich (St. Louis, MO, USA). DAC was dissolved in 0.45% NaCl containing 10 mM sodium phosphate (pH 6.8) and stored at −80°C, while ATRA was dissolved in absolute ethanol, protected from light and stored at −20°C. The concentration of the DAC and ATRA stock solutions was 100 μmol/l.

### Cells lines, cell culture and drug treatments

The human erythroleukemia K562 cell line was provided by the Jiangsu Institute of Hematology (Suzhou, China). The cells were cultured in suspension in Roswell Park Memorial Institute-1640 medium (Gibco, Grand Island, NY, USA) supplemented with 10% fetal bovine serum (Gibco) and incubated in standard tissue culture incubators at 37°C in a humidified atmosphere containing 5% CO_2_. Various concentrations of DAC and ATRA, alone or in combination, were added to the medium simultaneously. Briefly, the cells were treated with drugs as a sequential exposure for 24, 48 and 72 h using the following experimental conditions: 1 μmol/l DAC, 2 μmol/l DAC, 4 μmol/l DAC, 0.5 μmol/l ATRA, 1 μmol/l DAC plus 0.5 μmol/l ATRA, 2 μmol/l DAC plus 0.5 μmol/l ATRA and 4 μmol/l DAC plus 0.5 μmol/l ATRA. The untreated K562 cells were used as a control.

### Cell growth inhibition assay

Cell growth inhibition activity was determined using a water-soluble tetrazolium-1 (WST-1) cell proliferation and cytotoxicity assay kit (Beyotime, Haimen, China), according to the manufacturer’s instructions. Briefly, 100 μl cell suspension (containing 5×10^4^ cells) was plated in each well of 96-well plates. The cells were then treated with the two drugs as aforementioned for 24, 48 and 72 h. At the end of each experiment, the cell proliferation reagent, WST-1 (10 μl), was added to each well and the cells were incubated at 37°C for 4 h. The absorbance (A) at 450 nm of each well was measured using a spectrophotometer and the inhibitory rate was calculated using the following formula: Inhibitory rate (%) = [1 - (A_drug_ - A_blank_) / (A_control_ - A_blank_)] × 100. Three independent experiments were performed in quadruplicate.

### Cell differentiation and apoptosis assay

The expression of the myelomonocytic antigens, cluster of differentiation (CD)11b, CD14, CD13 and CD33, on the cell surface was determined by direct immunofluorescence staining and flow cytometry. Briefly, the cells were collected and washed with phosphate-buffered saline (Beyotime). A total of 5×10^5^ cells were stained with the following conjugated antibodies: CD11b-phycoerythrin (PE; catalogue number: 555388), CD14-fluorescein isothiocyanate (FITC; catalogue number: 555397), CD13-PE and CD33-FITC (catalogue numbers 555394 and 555626, respectively, BD Biosciences, Franklin Lakes, NJ, USA). The cells were incubated for 15 min at 4°C and then analyzed using a flow cytometer (FACScabilur; BD Biosciences). Apoptosis assays were performed using an Annexin V-FITC apoptosis detection kit (Beyotime) according the manufacturer’s instructions, and early apoptosis was evaluated by cytofluorometry (FACScabilur, BD Biosciences).

### Methylation-specific polymerase chain reaction (MSP)

MSP was used to determine the methylation status at the 5′CpG island in the p16 and RAR-β promoter regions. Bisulfite converts unmethylated cytosine residues to uracil, but methylated cytosines remain non-reactive. PCR amplifies uracil as thymine, while methylated cytosines are only amplified as cytosines. MSP distinguishes unmethylated from methylated alleles in a given gene based on sequence changes following bisulfite treatment of the DNA using primers designed for methylated or unmethylated DNA. The cells of different groups were collected for MSP at 48 h post-incubation. The DNA from the cell line was extracted using the ZR Genomic DNA II kit (Zymo Research Corporation, Irvine, CA, USA) as recommended by the manufacturer. Bisulfite modification of the genomic DNA was performed using the EZ DNA Methylation-Gold kit (Zymo Research Corporation) according to the manufacturer’s instructions. The PCR amplification was performed using p16 and RAR-β promoter gene fragment-specific primers for methylated or unmethylated DNA (Sangon Biotech Co., Ltd., Shanghai, China). The primers used for unmethylated p16 were: Sense, 5′-TTTTTGGTG TTAAAGGGTGGTGTACT-3′ and antisense, 5′-CACAAA AACCCTCACTCACAACAA-3′, which yielded a fragment of 132 bp. The primers used for methylated p16 were: Sense, 5′-GTGTTAAAGGGCGGCGTAGC-3′ and antisense, 5′-AAA ACCCTCACTCGCGACGA-3′, which yielded a PCR product of 122 bp. The primers used for unmethylated RAR-β were: Sense, 5′-TGGGATGTTGAGAATGTGAGTGATTT-3′ and antisense, 5′-CTTACTCAACCAATCCAACCAAAACA-3′, which yielded a fragment of 160 bp. The primers used for methylated RAR-β were: Sense, 5′-GGATTGGGATGTCGAGAACGC-3′ and antisense, 5′-CGACCAATCCAACCGAAACG -3′, which yielded a PCR product of 158 bp. The PCR was performed under the following conditions: 95°C for 4 min; 94°C for 25 sec, 62°C (p16) or 64°C (RAR-β) for 25 sec and 72°C for 30 sec for 25 cycles; and 72°C for 5 min. The CpGenome universal methylated DNA (Millipore Corporation, Billerica, MA, USA) was used as a control for the methylated DNA. The PCR-amplified products were separated by electrophoresis on 2% agarose gel and visualized by ethidium bromide staining under ultraviolet light. Images were then captured.

### RNA extraction and cDNA conversion

Following incubation with DAC and ATRA for 48 h, the cells were harvested for RNA extraction. Total RNA was extracted from freshly isolated culture cells, using the TRIzol one-step procedure (Invitrogen Life Technologies, Paisley, United Kingdom), according to the manufacturer’s instructions, and dissolved in diethylpyrocarbonate-treated water. Reverse transcription was performed using random hexamer primers for total RNA (2 μg/40 μl), and 100 units of MuLV reverse transcriptase (Fermentas, Hanover, MD, USA) was added to the reaction mixture, obtaining a significant enhancement of the assay sensitivity. The cDNA was stored at −20°C.

### Quantitative *PCR* (qPCR)

qPCR was performed using the 7500 Fast Real-Time PCR system (Applied Biosystems, Foster City, CA, USA) and all the primers were synthesized by Sangon Biotech Co., Ltd. The primers and probes specific for the p16 and RAR-β genes were as follows: p16 sense, 5′-CTGCCCGTGGACCTGGC-3′ and antisense, 5′-CTC TGGTTCTTTCAATCGGGG-3′; p16 TaqMan probe, 5′-AGT AACCATGCCCGCATAGATGCCG-3′; RAR-β sense, 5′-AGATCGTGGAGTTTGCTAAACGT-3′ and antisense, 5′-GGGTATACCTGGTGCAAATTCTAAG-3′; and RAR-β TaqMan probe, 5′-CAAATTACCCTGCTGAAGGCCGCC-3′. GAPDH was utilized as the housekeeping gene as an internal control of the RNA quality. The primers and probes used were as follows: GADPH sense, 5′-GGAAGGTGAAGGTCGGAGTC-3′ and antisense, 5′-CGT TCTCAGCCTTGACGGT-3′; and GADPH TaqMan probe, 5′-TTTGGTCGTATTGGGCGCCTG-3′. The reactions of the p16 and GAPDH gene amplification were performed under the following conditions: 95°C for 10 min; and 40 cycles of 95°C for 15 sec, 58°C for 40 sec and 37°C for 1 min. The PCR profile of RAR-β consisted of 95°C for 10 min, 10 cycles of 95°C for 15 sec and 60°C for 30 sec, followed by 40 cycles of 95°C for 10 sec and 58°C for 35 sec. The results were analyzed using ABI Prism 7500 SDS software (Applied Biosystems). The cycle threshold (CT) was determined and the differences in the CT values for p16, RAR-β and GAPDH were calculated. The expression of Genes with a CT of >35 cycles was considered absent. p16 and RAR-β expression was normalized to the simultaneously analyzed GAPDH. The comparative CT method was used to determine the relative expression levels of p16 and RAR-β, and the cycle number difference (ΔCT = CT p16/ RAR-β - CT GAPDH) was calculated for each sample. The relative p16 and RAR-β expression values are presented as 2^(-ΔCT)^. Each sample was measured in triplicate.

### Western blot analysis

For the western blot analysis, the cells were treated with DAC and ATRA for 48 h and then collected. Total protein from the cell line was extracted using cell lysis buffer for western blot anaylsis containing phenylmethanesulfonyl fluoride (Beyotime) according to the manufacturer’s instructions. The proteins were separated by 10% SDS polyacrylamide gel and transferred to nitrocellulose membranes. Following blocking with 5% skimmed milk, the membranes were incubated with an appropriate dilution of the rabbit anti-human polyclonal primary antibodies, anti-p16 (1:100), -RAR-β (1:100) and -GAPDH (1:1,000) (ABGENT, San Diego, CA, USA), followed by incubation with the horseradish peroxidase-conjugated goat anti-rabbit IgG secondary antibody (ABGENT), according to manufacturer’s instructions. The signals were displayed using an automatic film processor (SX435-T; Taixing Suxing Co., Ltd., Taixing, China). The signal intensity of the proteins was normalized against GAPDH using Quantity One software (Bio-Rad, Hercules, CA, USA).

### Statistical analysis

All experiments were repeated three times with similar results, and data are presented as the mean ± standard deviation. Data were analyzed using the one-way analysis of variance (ANOVA) and factorial design ANOVA. P<0.05 was considered to indicate a statistically significant difference. All statistical analyses were performed using the statistical software SPSS 17.0 (SPSS, Inc., Chicago, IL, USA).

## Results

### DAC and ATRA, alone or in combination, have no effect on the growth inhibition, differentiation and apoptosis of K562 cells

**Figure 1:**
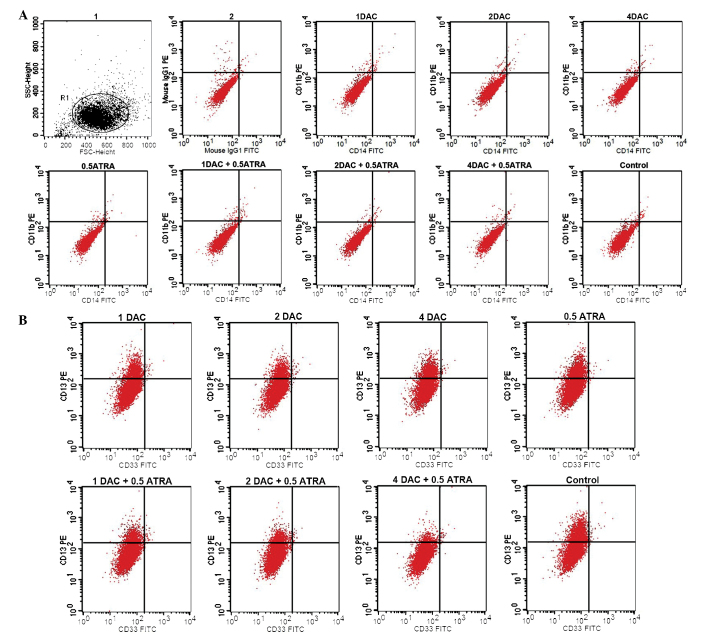
Outcome of DAC and ATRA on cell differentiation by flow cytometry. K562 cells were treated with the two drugs for 48 h. (A) The expression level of CD11b-positive cells was found in the top left and right gates and CD14-positive cells was found in the top right and bottom right gates. (B) The expression level of CD13-positive cells was found in the top left and right gates and CD33-positive cells was found in the top right and bottom right gates. DAC, 5-aza-2′-deoxycytidine; ATRA, all-*trans* retinoic acid; FITC, fluorescein isothiocyanate; PE, phycoerythrin.

**Figure 2:**
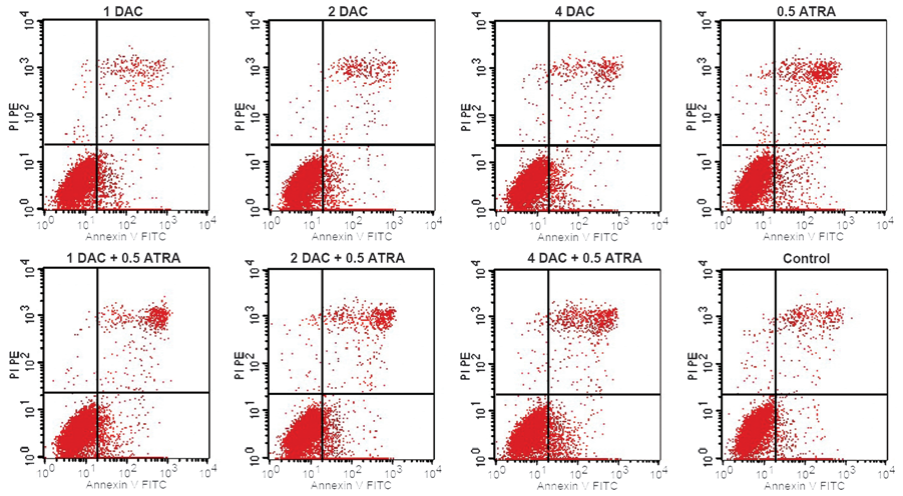
Effect of DAC and ATRA on the early apoptosis of K562 cells by flow cytometry. K562 cells were treated with the two drugs for 48 h. Apoptosis was assayed by Annexin V staining and fluorescence-activated cell sorting analysis. The early apoptotic cells (sorted only by Annexin V) are found in the bottom right gate. DAC, 5-aza-2′-deoxycytidine; ATRA, all-*trans* retinoic acid; FITC, fluorescein isothiocyanate.

The inhibitory rates of the drug-treated cells were all <20% (data not shown) and therefore, DAC and ATRA, alone or in combination, had no effect on growth inhibition (P>0.05). When the K562 cells were treated with the two drugs, no significant induction of CD11b or CD14 expression (typical markers of myelomonocytic differentiation of leukemia cells) was observed. The expression level of CD13 was 17.28±0.79% in the group treated with 1 μmol/l DAC plus 0.5 μmol/l ATRA, 12.56±0.81% in the group treated with 2 μmol/l DAC plus 0.5 μmol/l ATRA was and 5.45±0.76% in the group treated with 4 μmol/l DAC plus 0.5 μmol/l ATRA. These results indicated that the combination of DAC and ATRA was able to decrease CD13 expression compared with the control group (32.25±1.34%; P<0.05). The expression of CD33 was only faintly detected in the experimental groups (Fig. 1). CD13 and CD33 are markers of immature myeloid cells. DAC and ATRA, alone or in combination, had no significant effect on the early apoptosis of the K562 cells compared with the control group (P>0.05; Fig. 2).

### Analysis of the methylation of p16 and RAR-β genes in K562 cells

**Figure 3:**
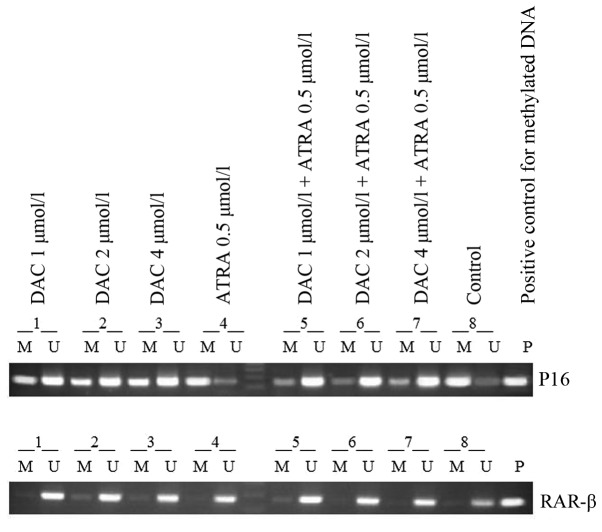
Methylation status of p16 and RAR-β promoters in K562 cells. M, methylated; U, unmethylated; p16, p16INK4a; RAR-β, retinoic acid receptor β; DAC, 5-aza-2′-deoxycytidine; ATRA, all-*trans* retinoic acid.

The MSP results showed clear signs of promoter methylation in the K562 cells for p16, and partial demethylation following treatment with DAC alone or in combination with ATRA, and the combined effect was more evident than that of treatment with DAC alone. The treatment with ATRA alone did not change the methylation status of the DNA, whereas the K562 cell line exhibited a band only when amplified with RAR-β primers for unmethylated and not for methylated DNA (Fig. 3).

### DAC in combination with ATRA activates the mRNA expression of the RAR-β gene in K562 cells

The mRNA levels of the p16 and RAR-β genes were detected by qPCR in K562 cells treated with drugs for 48 h. The results indicated that p16 was silenced by aberrant methylation in the K562 cells, and that DAC or ATRA, alone or in combination, were unable to re-express p16 by the demethylation of DNA. However, the RAR-β gene was unmethylated in the K562 cells, and the treatment of the cells with a combination of DAC and ATRA resulted in the upregulation of RAR-β. Following treatment of the cells with 1, 2 and 4 μmol/l DAC in combination with ATRA, the expression levels of RAR-β were 0.000003±0.000002, 0.000048±0.000015 and 0.000405±0.000093, respectively (P<0.05).

### Effect of DAC and ATRA on the protein expression levels of p16 and RAR-β in K562 cells

**Figure 4:**
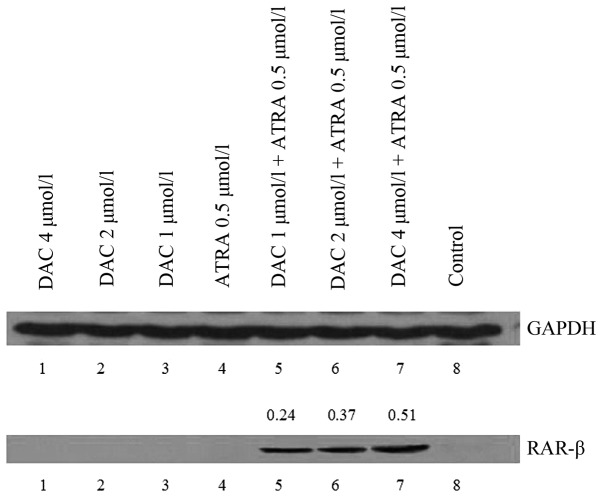
Effect of DAC and ATRA on the RAR-β protein expression of K562 cells. The signal intensity of the RAR-β proteins was normalized against GAPDH using Quantity One software. RAR-β, retinoic acid receptor β; DAC, 5-aza-2′-deoxycytidine; ATRA, all-*trans* retinoic acid.

Western blot analysis was performed on the K562 cells treated with DAC and ATRA for 48 h. No visible bands of p16 and RAR-β protein were detected in the untreated samples, however, RAR-β proteins were expressed in the drug-treated cells. Furthermore, strong bands were observed at higher doses of DAC in combination with ATRA (P<0.05; Fig. 4). The two drugs had no effect on p16 protein expression, which correlates with the changes in the mRNA level observed by qPCR.

## Discussion

Previous studies have reported that DAC and ATRA have the capacity to induce leukemic cell differentiation (10,12). Furthermore, DAC inhibits esophageal squamous carcinoma cell and thyroid carcinoma cell proliferation (13,14). The induction of apoptosis is of particular interest as a potential mechanism of action of the demethylating agents on the malignant and pre-malignant hematopoietic cells in leukemia. Aoyama *et al* (15) reported that the differentiating and apoptotic effects of DAC were dependent on the PU.1 expression level in PU.1-transgenic K562 cells. The current study demonstrated that DAC and ATRA have no effect on the growth inhibition, differentiation and apoptosis of K562 cells. However, the combination of DAC and ATRA was able to decrease CD13 expression. These observations indicated that the differentiating and apoptotic effects of DAC and ATRA in K562 cells may be associated with the regulation of the expression of certain genes.

The pathogenesis of myeloid malignancies involves epigenetic changes, and efficacy has been shown by hypomethylating agents in these diseases. The methylation of the 5′CpG island in the p16 and RAR-β genes is associated with the transcriptional silencing of the gene in a number of neoplasms, including solid tumors and leukemias (16). Uenogawa *et al* (17) reported that azacitidine induces the demethylation of p16 in adult T-cell leukemia/lymphoma. In the current study, the aim of the MSP analysis of p16 and RAR-β in the K562 cells was to assess the methylation status of the promoter in the genes. The results showed clear signs of promoter methylation in the K562 cells for p16, and partial demethylation was evident following the treatment of the K562 cells with DAC alone or in combination with ATRA, but more evidently for the combined effect. ATRA alone had no effect on methylation and the RAR-β promoter region was not methylated in the K562 cells. The K562 cells did not express p16 and RAR-β, however, the expression of RAR-β was upregulated by treatment with DAC in combination with ATRA for 48 h. A higher expression of RAR-β was observed at higher doses of DAC in combination with ATRA, but the two drugs had no effects on p16 expression. These results indicated that DAC induces p16 promoter demethylation, however, the demethylation does not affect the mRNA and protein expression of p16. The RAR-β gene was unmethylated in the K562 cells, however, DAC in combination with ATRA induced the expression of the tumor suppressor gene, RAR-β, with unmethylated CpGs in the K562 cells. Therefore, RAR-β may be one of the target genes of the two drugs in acute erythroleukemia. We hypothesized that DAC upregulates RAR-β gene expression in K562 cells, not by demethylation, but by any other mechanism. However, the precise mechanism by which DAC in combination with ATRA increases RAR-β expression in K562 cells has not been well defined.

In conclusion, DAC induces p16 promoter demethylation, and ATRA enhances this effect. The two drugs synergistically activate RAR-β expression, which indicates that DAC used in combination with ATRA has clinical potential in the treatment of human erythroleukemia.
